# The athletic work force: Sport as a key to employment for people with intellectual disabilities?

**DOI:** 10.3233/WOR-220330

**Published:** 2023-12-15

**Authors:** John Selander, Erika Wall

**Affiliations:** a Rehabilitation Science, Department of Health Sciences, Mid Sweden University, Sundsvall, Sweden; b Sociology, Department of Health Sciences, Mid Sweden University, Sundsvall, Sweden

**Keywords:** Sports, interview, surveys and questionnaires

## Abstract

**BACKGROUND:**

People with disabilities are employed at lower rates than non-disabled individuals and, among people with disabilities, those with intellectual disabilities have most difficulty finding and keeping employment. The reasons for the low labour participation among people with intellectual disabilities are many. Sport participation has a number of positive effects for the individual, and it is reasonable to hypothesise that sport participation favours labour-force participation for individuals with intellectual disabilities.

**OBJECTIVE:**

The dual aim of the current study was to investigate labour market participation among Swedish athletes with intellectual disabilities attending Special Olympics Invitational Games, and to investigate these athletes’ experiences regarding the effect of sport participation on finding and keeping a job.

**METHOD:**

The study design includes two parallel data collections, a survey and an interview study. The survey was analysed using descriptive statistics and the interviews were analysed using content analysis.

**RESULTS:**

The major result of the survey was the large number of individuals with intellectual disabilities who were in work: among men, 72% and, among women, 44%. This result was encouraging and differs from previous statistics on employment among Swedes with intellectual disabilities. The content analysis resulted in a first step in the categories *manual work*, *individual sports* and *team sports*, and in a second step, where the relation between sports and work was analysed, in two categories, namely *indirect and direct relations between sport and work*.

**CONCLUSION:**

To improve chances for individuals with intellectual disabilities to find and keep a job, sports participation should be encouraged.

## 1 Introduction

Around the globe, people with disabilities are employed at lower rates than non-disabled individuals and, among people with disabilities, those with intellectual disabilities (ID) have most difficulty finding and keeping employment [1, 2]. The reasons for the low labour participation among people with ID are many and found at different levels in society, i.e., societal, organizational, and individual. Previous research, with no specific focus on ID [3], shows that sport participation has a number of positive effects for the individual. In addition to physical benefits, sport participation has positive psychological and social effects, e.g., self-confidence, self-efficacy, and general well-being, all factors of high relevance for finding and keeping a job. Against this background, it is reasonable to hypothesise that sport participation also favours labour-force participation for individuals with ID.

### 1.1 Background

Employment is a crucial part of life and is, for the individual, associated with several important domains, such as strengthened identity, better economy, improved self-confidence, access to social networks, better health, and improved life satisfaction [4, 5]. However, despite civil rights and legal efforts around the world, individuals with disabilities are employed at lower rates than individuals without disabilities. Research shows that this is a global phenomenon [1], with Sweden being no exception. In Sweden, the employment rates for working-age individuals without disabilities are 83% for men and 80% for women, while corresponding figures for individuals with some sort of disability are 66% and 61% respectively [6]. Employment rates among individuals with disabilities differ greatly depending on the type of disability [7] and for individuals with ID the employment rates in Sweden are 27% for men and 16% for women [8]. These figures are also in line with other European countries [9].

The barriers for employment experienced by individuals with ID are many and found at different levels [10, 11]. At a societal level are found laws and regulations, but also norms and attitudes [12]. In a review study by Nardodkar et al. [13], where the focus was on laws and regulations in UN member states (in total 194 countries), it was found that, nearly 50 years after adoption of the International Covenant on Economic, Social, and Cultural Rights, many states lack laws that prohibit discrimination against persons with ID during recruitment and employment. The authors concluded that legal discrimination against persons with ID continues to exist. When it comes to attitudes, studies identify numerous negative attitudes that strongly contribute to stigmatisation. In the hierarchy of social acceptance for individuals with disability, those with ID are consistently found to be the least socially accepted and the most socially excluded and stigmatised [14]. Employers consider those with ID neither socially nor professionally competent [15]. On an organisational level, the laws and regulations are set in a practical context, resulting in decisions and practices with direct impact on the individual. At this level, we also find employer and co-worker attitudes. Studies show, for example, that employers with experience with ID, either in private or professional life, have more positive attitudes about hiring people with ID than employers without such experience [10, 16]. At the individual level, finally, we find specific individual prerequisites, such as personality, gender, work ability, social skills, wishes, and ambitions [17], and also the individual’s private sphere, for example family and friends and their attitudes and support. Studies show that family support is essential for individuals with ID to find and keep a job [18], and that over-protective parents sometimes are a problem [19]. To understand the barriers to the labour market experienced by individuals with ID, one must both consider factors at different levels and how these different factors interact [9].

### 1.2 Intellectual disability and sport participation

People with ID are less physically active, less physically fit, more sedentary, and more likely to be overweight or obese than the general population [20–23]. Individuals with disabilities, and especially with ID, participate more seldom in organised sports [24]. Taken together, this constitutes a problem, since physical activity and sport have several positive effects for individuals with ID as for individuals without disabilities [25, 26]. In addition to physical benefits, physical activity and sports skills appear to benefit both psychological and social health and general quality of life in individuals with ID. Further, a comprehensive longitudinal study by Kaari et al. [27] (with no focus on ID), examined the role of physical activity and sport during youth for later labour market participation, and physical activity and sport participation were found positively related to the probability of being employed. The study concluded that investments in childhood physical activity and sport may not only promote health and well-being, but also correlate with better labour market outcomes later in life, providing both personal and societal benefits.

In summary: Labour market participation among individuals with ID is low. Individuals with ID are less physically active and participate in organised sports less often. Sport participation has numerous positive outcomes related to physical, mental, and social wellbeing, and general quality of life. For the general population, sport participation correlates with better labour market outcomes. It is reasonable to hypothesise that sport participation facilitates finding and keeping a job for individuals with ID, as well.

Against this background, it is of interest to explore labour market participation among physically active athletes with ID. Thus, the dual aim of the current study is to investigate labour market participation among Swedish athletes with ID attending Special Olympics Sweden Invitational Games (SOSIG), and to investigate these athletes’ own experiences regarding the effect of sport participation on finding and keeping a job.

### 1.3 Special Olympics Sweden Invitational Games (SOSIG)

In 1968, the Special Olympic (SO) movement was founded by Eunice Kennedy Shriver to involve persons with ID in sports training and competition. Over the years, SO have been held every second year in various countries, first solely as summer games, but, since 1977, also as winter games. Today, SO is the largest sports event in the world serving athletes with ID. The last SO winter games were held in Austria, in 2017, with around 2,700 athletes from 107 nations competing in nine different sports. The subsequent SO winter games were planned for 2021 in Östersund, Sweden, but were cancelled for economic and political reasons. A planned pre-game competition, called Special Olympics Sweden Invitational Games (SOSIG), with a smaller number of invited athletes, did however take place. This was held in Östersund, February 2020, with a total 450 athletes from 19 different nations participating. Of the total 450 athletes, 145 were Swedes. The different sports in the SOSIG 2020 games were downhill skiing, floor hockey, snowshoe running, figure skating, cross-country skiing, short track skating, and snowboarding. To get a picture of the event, one should bear in mind that SOSIG is more a fun and social sport gathering for young adults with ID, than a traditional competitive sport event. The athletes are with few exceptions very dedicated, but a vast majority are not top athletes.

## 2 Materials and methods

### 2.1 Study design

The study design includes two parallel data collections, a survey and an interview study, both related to the SOSIG in Östersund, Sweden, January 2020. The survey aimed to gain a picture of the Swedish participants’ labour force participation, while the interviews aimed to include the voice of the athletes themselves, and to gain understanding on how athletes with ID view the relationship between work and sport.

### 2.2 Survey

#### 2.2.1 Recruitment & participants

The researchers presented their aim to the organisers, i.e., SOSIG, who supported the idea and agreed to help with the planned study. For ethical reasons, the authors and SOSIG agreed that all information from the authors should be delivered by SOSIG. Thus, athletes and their leaders remained anonymous for the authors.

A short digital survey, aimed for the in total 76 leaders who accompanied the 145 Swedish athletes participating in the SOSIG games, was constructed by the authors and put online in February 2020. The leaders (e.g., a parent, relative, or sport coach) were the athletes’ contact persons in relation to SOSIG, and information about the survey was delivered by SOSIG directly to the leaders. The leaders were, in most cases, responsible for one or two athletes, but leaders were also responsible for teams of athletes (up to 11 athletes). The survey included questions regarding the athletes’ sex (men/women), age (0–20/21–30/31–40/41–50/50+), employment situation (not in work/in work part-time/in work full-time), type of salary (wage subsidy/regular salary/do not know), type of employment (private/public).

#### 2.2.2 Survey analysis

The survey was analysed using descriptive statistics.

### 2.3 Interview study

#### 2.3.1 Recruitment & participants

Participants were recruited through the SOSIG organisation and limited to athletes diagnosed with ID. For ethical reasons, a contact person at SOSIG informed participating leaders about our study, and that the authors wished to conduct interviews with athletes with ID who had a job. A “job” was defined as some sort of paid work (wage subsidy included, sheltered work/daily activity excluded). Those interested in participating were asked to contact the first author. In total, we were contacted by 14 leaders, which, for different reasons, resulted in interviews with seven athletes. All interviews were audio-recorded and transcripted verbatim.

#### 2.3.2 Interview guide & interviewing

All interviews were performed as follows. Firstly, researchers met the interviewee and, if appropriate, his/her leader and/or assistant at the official meeting point for the SOSIG (which was the Mid Sweden University campus). Together, the pair or group went to a smaller separate room. Before the interview began, we reviewed the consent previously provided via email and solicited consent a second time, and after a description of the layout and content of the interview, the interview itself begun.

All interviews followed the same *interview guide*. Initially, short background questions were asked, followed by questions on *sports*, in which interviewees were asked as well about participating in the SOSIG, as well as participation in other sports. Then, questions were asked about *work* and participation in wage labour. Questions concerned current work as well as any previous work. Both topics of the interview guide included questions about social relations in sports and work. Lastly, respondents were asked about their thoughts on the potential relationship between sports and work, or, more precisely, the role of sports in finding and keeping a job.

In total, seven interviews of 17— 32 minutes were made. Four interviews were led by first author and three by the second author. Four respondents participated alone and three were accompanied by a leader.

The interviews were strictly delimited to questions on sports and labour force participation. That is, interviews were made with the participants in their roles as professionals. No personal questions were asked, nor questions about health in general or the interviewee’s specific ID. However, based on observations during the interviews, the participants can be described as of various ages with various types and degrees of intellectual disability.

#### 2.3.3 Interview analysis

To arrive at an understanding of how the athletes with ID experienced combining sports with work, descriptive content analyses [28] focusing on what was said by the participants (manifest analysis) specifically on the predetermined themes on work and sports respectively, and the relation between work and sports, was carried through. With this directed approach to content analysis, the goal was to validate and deepen the knowledge from the statistical analysis. The content analyses were made in a two-step process: firstly, the characteristics of sports and work were analysed and described; secondly, the *relationship* between sports and work, and especially the respondents’ thoughts regarding sport as a potential facilitator for finding and keeping a job, was investigated, and described. Initially, the material was read by the two researchers who both marked meaning units within the material, that is statements relating to the same central meaning [28], in this case sports and work respectively. The meaning units where then condensed, coded and thereafter interpreted as described by Graneheim and Lundman [28]. From this, categories were created reflecting the predetermined focus of sports, work and the relation between sports and work.

### 2.4 Ethics

Research (data collection, handling of data, analysis, reporting of results) was designed in accordance with the guidelines of Swedish Research Council. In this case, data collection does not include questions on personal information referred to in the regulation and the research is therefore not covered by the Swedish formal legislation on ethical review. However, from an ethical viewpoint, it’s crucial to handle the study with humility. People with ID have most often been included within research via someone speaking on their behalf (professionals, support workers, or parents) [29]. We agree with researchers of the disability rights movement, emphasising that it is unethical to assume those with ID cannot speak on their own behalf (see for example 30–32). Therefore, we combine information about athletics with ID (through a survey with leaders) with interviews with the athletes themselves. The narratives of those included in qualitative research might have a positive impact on the lives of others with ID [33, 34]. However, one must remember the long and difficult history of unethical research [35, 36] including abuse, discrimination, and exploitation [37] making research including people with ID sensitive even today.

## 3 Results

### 3.1 Survey

After one reminder, the survey was completed by 48 of the total 76 leaders, yielding a response rate of 63%. Further, the 48 leaders who completed the survey represented 124 of the total 145 Swedish athletes who participated in the games, yielding information about 86% of the Swedish athletes attending. This sample (*n* = 124) consisted of 83 (67%) men and 41 (33%) women. Among men, 91% were aged 40 or younger. The corresponding figure for women was 95%.

Among men, 20 (24%) were not in work, 26 (31%) had part-time work, and 34 (41%) had full-time work. Among women, 23 (56%) were not in work, 14 (34%) worked part-time and 4 (10%) worked full-time. Regarding salary, 69 (83 %) of male athletes and all of the female athletes received wage subsidy/government disability subsidy. Accordingly, 14 (17%) of the male athletes received a regular salary from the employer. Regarding type of employment, 71% of men worked in the private sector and 29% in public sector. Among the women, the division was 46% and 54% respectively.

### 3.2 Interviews

The seven respondents all combined sports with wage labour. When it comes to work history, their experiences ranged from two months to ten years. Regarding sports, several respondents combined various sports, but competed in one particular.

#### 3.2.1 Work and sports

The first step of the analysis gives background information on work and sports among the interviewees. Hence, the results on work and sports respectively describe the content of the material collected in the interviews.

Regarding **work**, the content analysis shows that respondents had various kinds of work, whereas all work are included within the same analytical category, namely *manual work*, as illuminated in Table 1 (below). The category consists of two types of work: *blue-collar jobs* and *service jobs* respectively. Blue-collar job reflects that some participants describe themselves as factory workers, attendants, fitters, or cleaners. Also, two respondents worked as assistants at a hospital and a restaurant, here in defined as service jobs. Several participants report having full-time work while others work part time. None was employed less than half time.

**Table 1:** Analytical categories, and sub-categories created from the content analysis on work and sport respectively

Category	Manual work	Sports
Sub-categories:	•Blue-collar work	•Individual sports
	•Service jobs	•Team sports

When it comes to **sports**, most respondents have experience practicing various sports. From this, *individual sports* and *team sports* occurred in the analysis. The following individual sports are mentioned in the data: orienteering, running, athletics, snowshoeing, cross-country skiing, badminton, bowling, and riding. The team sports mentioned in the material were floor hockey, soccer, and handball as summarized in Table 1. In the current competition, however, all participated in just one sport. The respondents’ involvement in sports varied, but sports played a central role in the lives of all. On a weekly basis, time spent on physical training ranged between four and twelve hours. Further, involvement in competition varied among the individuals.

#### 3.2.2 The relation between sport and work

In the second step of the analysis, the relation between sports and work were analysed. The results presented are followed by a table summarizing the categories and sub-categories created from the content analysis (see Table 2, below).

**Table 2:** Analytical categories, and sub-categories created from the content analysis on the relation between sport and work

Category	Indirect relations	Direct relations
Sub-categories:	•Social aspects	•Formal public support
	•Psychologically strengthening	•Private contacts
		•Personal advantages
		•Resources
		•Disadvantages

The importance of the respondents’ involvement in sports, and their appreciation, were evident from the interviews. Regarding which aspects of sport engagement were most important, all pinpointed sport’s social aspects, i.e., they have friends in the sport community and the sporting community (especially fellow athletes) was like a second family. The importance of leaders in sports was also mentioned as important. The social aspects of attending in sports indirectly contributing to the relation between sports and work constitutes the first category in this part of the analysis: **indirect relations between sport and work**. This first category is defined by how the relation between work and sports is understood as indirectly connected where social relationships in sports is found facilitating the possibility of getting a job and the physical strength from engaging in sports is contributing to the possibilities fulfil the duties at work.

Firstly, the indirect relations between sport and work were found in relation to *social aspects*, the first sub-category:

Sport brings a lot of happiness, new friends, a sense of belonging. The team is like a new family. (IP1)

The best part of sport is that it’s fun. A blast! I hang out with two athlete friends in my spare time. (IP6)

Also, competition was mentioned in the interviews, and travel.

The best part is travelling to competitions. The happiness from travelling. (IP1)

Competition is my favourite. Running races in the summer. Travel is the best part of sport. Travelling abroad! (IP7)

Beside its social aspects, interviewees highlighted, e.g., that sport is *psychologically strengthening*, what constitutes the second sub-category here.

You grow as a person, and we aren’t so fragile. We’re stronger than others think. (IP1)

The best part of sport is that it gives self-confidence. You can do stuff. (IP2)

In addition to sport’s more indirect positive aspects, in finding and keeping a job, more **direct relations between sport and work** were mentioned. This second category on the relation between sports and work is defined by how the relation between work and sports is understood as directly connected through formal public support and private contacts enabling job, and through personal advantages and resources strengthen the position in work but also some disadvantages on the difficulties combining sports and work. This category constitutes of several sub-categories reflecting the various perspectives on how sports was found as facilitating the possibilities finding and/or keeping a job: formal public support, private contacts, personal advantages, resources but also disadvantages combining sport and work (for an overview of the analysis, see Table 2).

In the interviews, participants were asked about their introduction to the labour market. In most cases, *formal public support* was highlighted as crucial for getting job, what constitutes the first sub-category here. In the material, internship during schooling (IP 5, IP7), a public-supported project for people with ID (IP4), and the Swedish Public Employment Service (IP2) were highlighted as crucial for getting a job, as in the following quote:

This [my job] I got …  , yeah, I got it through the Public Employment Service. (IP2)

*Private contacts,* the second sub-category, were also pinpointed within the material, as some interviewees described getting their job through social contacts. In one case, the leader was described as the key person in seeking employment (IP6). In the present study, the relation between sports and work was at focus. Thus, *private contacts* in the sports were described as a crucial facilitator in finding a job in two of the seven interviews.

In the following quote, the interconnection between private social contacts and sports is highlighted. The interviewee recounted that “we knew people” and that “it was probably Dad who knew someone”, in both cases in the context of sports.

Yeah, [I worked with] the sports movement’s study association a while, helping out. And yeah, it was because we knew people. That’s how we got in. Then it was the same way at Parasportförbundet— it was Dad who knew someone. (IP2)

In the next example as well, the interrelation between sports and social relations is highlighted. Here, the respondent described how social contacts from a sports camp led to possible interest in a summer job at a factory. Experiences working extra led to a probationary employment (and, later on, a permanent position):

*Question:* Do you remember how you got the job? *Answer:* Sure, it was a …  . Someone I knew [the son of a manufacturer whom IP met at orienteering camp], who …  . Or, rather, I was there …   the guy who owns the factory, his son was there when I was at camp, when I like, if …  . ’Do you want a summer job at our factory?!’ And the first three summers, before I started working, I had a summer job there. Then I applied …  that’s how I started working there. And then I got a probationary position. (IP1)

When asked about sport in relation to keeping a job, and any possible advantages and/or disadvantages of combining sport and employment, several specific examples were mentioned in the material.

Regarding *personal advantages*, the third sub-category describing the relation between sports and work, illuminating the relationship between sports and work, four of the interviewees pinpointed that the physical strength that they get from exercising provides benefits at work, as highlighted in this quote:

A bit, yeah, actually. A bit. Since it’s …  . Sometimes it’s physically demanding …  . Then it can be good to have done weight-training, I mean, for example. (IP4)

I’ve got a tough job, but I manage because I am strong. It’s smelly and crowded at work, that’s why so many quit. The boss is really satisfied with me. I’m good at carrying sacks. Sport has made me strong. (IP4)

Actually, the importance of physical strength was in one case described as necessary to doing the job:

If someone …  I didn’t …  like, if I hadn’t exercised at all, then I don’t think I could have …  had this job. (IP1)

I stand up at work all day, and I couldn’t manage that if I didn’t do sport. (IP3)

As described, the interviewees worked within in blue-collar jobs and service, that is, physically demanding work.

Further, combining sport and work seems a question of *resources*, which constitutes the fourth sub-category. To get paid is described as important for sport, but, at the same time, sport is described as most essential, before work. That is, the resources from a job create the conditions for sports and competition. As illustrated by the following quotation, work enables focusing on sports:

If I got money without having to work and just spent time doing sport, then …  I mean, my job I do to get to go on …  for example, I could go on this [Special Olympics]. That I got to, like, make money. We don’t get paid …  to be here. But if there was the possibility, to get paid for doing your sport, it would have been priceless for me. Since, then, you could spend much more time on sport. You could spend a lot more time on individual workouts. (IP5)

This respondent stated that it would be preferable to have sufficient resources to enable focusing on sports exclusively:

No, I mean, I can say it like this: If I could do sport without having to work, that would be …  . If I could live off sport, then I would do it. But, as it is now, it’s not possible. That’s how it is now …   (IP5)

In the material, some *disadvantages* related to combining sport and job also emerged. These problems, which constitutes the last sub-category, were relating to combining these two activities. Such disadvantages were related to endurance, time, and resources.

One of the interviewees mentioned that it can be difficult to exercise in the evening, despite a longing to run, due to lack of energy. In this case, a lunch break was used for training to enable combining sport and work:

It feels like I have to, like, get out and move. Often during my lunch break I go out and run …  a loop through the forest nearby. If I have time. Otherwise …  when I come home later, I usually feel exhausted when I come home. (IP1)

In the quotation above, not only is endurance mentioned, but also the difficulties creating time for both sport and work. Time was repeatedly a problem, not only difficulties having time to combine sport and work, but also the consequences of combining sport and work on other free time activities. When sport and work take all available time, little time is left for anything else, as emphasised by IP3. Similarly, in the quote below, the respondent stated that he is away until the late evening, making it difficult doing anything else during his spare time:

I work from eight to five. Afterwards, I work out …  or, after work, I go— pretty stressed— to the sport hall to work out, and then you don’t get home before nine or ten, and you don’t have much extra time. I usually talk with my girl then, otherwise it doesn’t happen much. (IP5)

In summary, the analysis based on the interviews with Swedish Special Olympic athletes with ID show that participation in sports have number of positive effects in life in general, and, particularly regarding the relation between sport and work, when it comes to finding a and keeping a job. Some respondents got their job through formal contacts, while others refer to personal contacts, and, for some, sport contacts. Some interviewees find their physical strength, acquired through sports, to be important in performing their work, while others highlight the importance of resources derived from work. Also, some difficulties regarding combining sports and work were found. Such disadvantages relate to the possibilities combing exercise and working (endurance, time).

## 4 Discussion

The major result of the survey was the large number of individuals with ID who were in work: among men, 72% and, among women, 44%. This result differs from previous statistics on employment among Swedes with a disability. Those reporting a disability have employment rates of 66% (men) and 61% (women) respectively [6], and 27% and 16% for those with ID [8]. Even though these samples differ from our sample in different ways, e.g. regarding age, our finding, that so many athletes with ID are employed, was both surprising and encouraging.

How, then, can the high rate of labour market participation among athletes be explained? Is there causality, i.e., does involvement in sport increase the chances of finding and keeping a job? Or is the relationship explained by other factors, i.e., selection? The results from the survey in the current study give no answer to these questions, but it is reasonable to believe that there is a selection, i.e., individuals with ID who are involved in organized sports (also not on elite level) more often suffer from relatively mild problems and differ from individuals with ID who are not in terms of, e.g., physical and mental health, and social support. Thus, their high rate of labour market participation partly is explained by these and other resources that the athletes possess. But, from our interviews, it is also reasonable to assume that participation in sport contributes and strengthens the resources necessary for finding and keeping a job. Regarding finding a job, the content analysis illuminated the importance of private contacts, often within the sport community. One can assume that a perception that private contacts were crucial for getting a job implies that the employers in question are aware of what hiring individuals with ID entails. That is, these results, related to the process getting hired, can be related to previous research [10, 16], which shows that employers with experience with ID are more positive about hiring people with ID. Formal public support was likewise highlighted as important in analysis, confirming previous research that pointed out that financial support was found to be a helpful side by side with awareness-raising activities and non-financial support [38]. Regarding the importance of formal public support, one must remember that the results relate to the context of Sweden, which has a tradition of strong social support for private as well as public employers of people with ID. Our interviews also indicate sport participation as positive for keeping a job. For example, physical strength related to sport appeared in the content analysis as important for work. Such reasoning resembles the analysis of young blue-collar workers perception of their own commitment to sports as well as manual labour [39]. That is, growing up in working-class conditions is understood to contribute to a positive relationship between physical activity and manual labour. From this, we can argue that the interrelationship between work and sport might not be specific to employees with ID but an expression of general mechanisms. In the interviews, the benefits of physical fitness were expressed in various contexts. In addition to physical strength, the importance of psychological and social resources deriving from work are highlighted from the content analysis. These findings all agree with a great amount of research regarding positive outcomes from physical activity and sport, regardless of disability status, when it comes to resources that contribute to quality of life.

The findings regarding differences between the sexes, i.e., that more men than women with ID are employed, that men work full time and women part time, and that men are employed in the private and women in the public sector, are in line with previous findings [8, 40]. It is nevertheless interesting to consider them. One interesting hypothesis, discussed by Lövgren [1], has to do with an effort among individuals with ID to conserve traditional gender roles. In her thesis, she argues that men and women with ID have long been considered a ”gender neutral group”, and that the individuals in her study, in reaction, intentionally exaggerate differences between the sexes. In this sense, it is preferable to fit in to a traditional role, for men and women, then to belong to the gender neutral and more diffuse group of “individuals with ID”. Perhaps with this hypothesis are the differences in our study to be explained, i.e., more men than women are part of the labor force, and men working full time in the traditionally male-dominant private sector while women work part time in the traditionally female-dominant public sector.

Finally, those interviewees defining themselves foremost as athletes see employment as a tool to earn money to focus on sports but, simultaneously, as a hinder regarding time, even if employment was appreciated. The lack of spare time experienced when combining full-time work with sport is not surprising, and probably a problem that athletes with ID share with athletes without disabilities who combine sport and full-time work. However, it is reasonable to believe that workers with ID must invest relatively more effort, energy, and concentration on their work than employees without disabilities. This makes combining work and sport a greater problem. These results highlight the difficulties of being recognised as an athlete with ID, which should be further studied to ensure the conditions for equal sport participation.

### 4.1 Limitations

This study used materials from a survey and an interview study. However, it should be remembered that both sources are specifically related to the Special Olympics Invitational Games in Östersund 2020. Also, since the data collected from the survey was limited, and relatively few interviews were made, conclusions from the study should be drawn with caution. The statements presented in the interviews shall be seen as expressions of subjective experiences regarding sport and jobs. However, we mean that these experiences are important to illustrate how individuals with ID view the relation between their working lives and their sport activities. The survey was answered by the leaders of the athletes. We had no method to ensure that the leaders reported truthful information, but since the questions were few and strictly focused on the demographic and employment characteristics of the athletes, we have no reason to believe that information given was false. Finally, one should bear in mind that the participants included in this research in different aspects differ from individuals with intellectual disabilities in general.

## 5 Conclusion

Labour market participation among individuals with intellectual disability is low, but among those who participate in organized sports, labour market participation is higher. Interviews with individuals with intellectual disabilities indicate that participation in sport strengthens resources necessary for finding and keeping a job. To improve chances for individuals with intellectual disabilities to find and keep a job, sport participation should be encouraged, not only by family and friends, but also by professionals working with this group (e.g. habilitation, school). In addition, sport organizations need to better include individuals with intellectual disabilities in regular activities.
